# Sinapic acid prevents adipogenesis by regulating transcription factors and exerts an anti-ROS effect by modifying the intracellular anti-oxidant system in 3T3-L1 adipocytes

**DOI:** 10.22038/IJBMS.2022.62590.13847

**Published:** 2022-05

**Authors:** Cordelia Mano John, Sumathy Arockiasamy

**Affiliations:** 1 Department of Biomedical Sciences, Faculty of Biomedical Sciences and Technology, Sri Ramachandra Institute of Higher Education and Research, Porur, Chennai – 600116, Tamil Nadu, India; 2 Department of Biomedical Sciences, Faculty of Biomedical Sciences and Technology, Sri Ramachandra Institute of Higher Education and Research, Porur, Chennai – 600116, Tamil Nadu, India

**Keywords:** 3T3-L1 adipocytes, Adipogenesis, FAS, PPARγ, ROS, Sinapic acid

## Abstract

**Objective(s):**

In this study, we tested the hypothesis that sinapic acid (SA), a naturally occurring hydroxycinnamic acid found in vegetables, cereal grains, and oilseed crops with various biological activities suppresses adipogenesis in 3T3-L1 adipocytes by down-regulating adipogenesis transcription factor.

**Materials and Methods:**

3T3-L1 adipocytes were treated with SA and evaluated by Oil Red O staining, triglyceride estimation, lipolysis, and reverse transcription-polymerase chain reaction. 3T3-L1 adipocytes were treated with various concentrations of SA (100 to 1000 μmol) during differentiation.

**Results:**

SA prevented an increase in adipocytes by reducing preadipocyte clonal expansion. ORO staining analyses revealed that SA reduced cytoplasmic lipid droplet accumulation in 3T3-L1 by 57% at the highest concentration of 1000 μmol without affecting cell viability. Furthermore, SA down-regulated the expression of peroxisome proliferator-activated receptor-gamma, CCAAT/enhancer-binding protein alpha, sterol regulatory element-binding protein 1c, and fatty acid synthase. ROS generated during adipogenesis was also attenuated by SA treatment by increasing antioxidant enzymes superoxide dismutase, catalase, and the cellular antioxidant glutathione. SA demonstrated no in vivo toxicity in the Drosophila melanogaster model.

**Conclusion:**

These results suggest that SA exerts anti-oxidant and anti-adipogenic effects and could be used as a functional nutraceutical ingredient in combatting obesity-related diseases.

## Introduction

Obesity is a chronic disorder with a complex etiology linked to many health complications. It is characterized by excessive accumulation of fat in the body as a result of an imbalance in energy intake and consumption, and it is often linked to conditions like diabetes, hypertension, hyperlipidemia, and cardiovascular diseases (1). The impact of obesity on worldwide public health has increased in recent years, with a global incidence of 39% (2). The currently approved anti-obesity drugs involved are associated with many adverse effects like increased heart rate, constipation, flatulence, bloating, dry mouth, fatty stools, dyspepsia, nausea, insomnia, and diabetes mellitus (3). 

However, obesity is intimately linked to aberrant adipose tissue expansion and accumulation, which is mostly due to excessive preadipocyte differentiation and adipocyte hypertrophy. Adipocytes play an important role in energy homeostasis by increasing in size (hyperplasia) and number (hypertrophy) and storing it as an intracellular triglycerides. The rate of adipogenesis is very closely related to obesity (4). The transcription factors network of peroxisome proliferator-activated receptor-gamma (PPARγ), CCAAT/enhancer-binding protein beta (C/EBPβ), and sterol regulatory element-binding protein-1c (SREBP-1c) stimulate adipogenesis and lipogenesis, two interconnected processes that link preadipocyte differentiation and fatty acid biosynthesis (5, 6). Lipogenesis is triggered by SREBP-1c which alters the expression of lipogenic gene fatty acid synthase (FAS) (7). On the other hand, lipolysis is initiated by the enzyme, hormone-sensitive lipase (HSL), which breaks down intracellular triglycerides and diacylglycerols to release free fatty acids and glycerol (8). Moreover, dysfunctional adipogenesis is linked to generation of reactive oxygen species (ROS) in cells *via* NADPH oxidase 4 (NOX4) and vice versa (9). 

**Figure 1:**
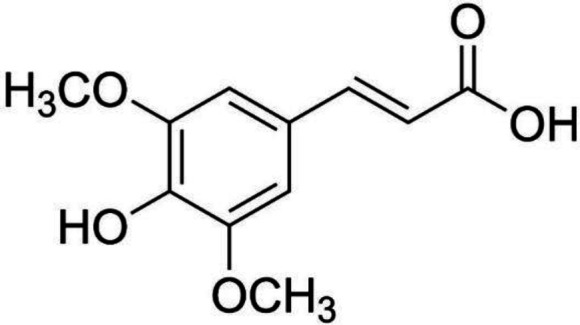
Chemical structure of sinapic acid

Polyphenols exhibit biological activities such as anti-oxidant, antimicrobial, anti-inflammatory, anxiolytic, hepatoprotective, cardioprotective, and anti-carcinogenic (10). Sinapic acid (SA; 3,5-dimethoxy-4-hydroxycinnamic acid), is a cinnamic acid polyphenol with ring substitutions of two dimethoxy groups at the 3^rd^ and 5^th^ and one hydroxy group at the 4^th^ position of the phenyl group (Figure 1). SA is abundantly found in cereals, spices, vegetables, citrus fruits, berries, and oilseeds (11). Previous studies have suggested the anti-obesity effect of hydroxycinnamic acids, such as ferulic acid (12), caffeic acid (13), and *p-*coumaric acid (14). SA is known to possess several beneficial properties against oxidative stress (11), inflammation (15), and diabetes (16). 

In dissatisfaction with the high cost and harmful side effects of obesity drugs, the current research has led to natural products as an excellent alternative for treating obesity and gained widespread momentum (17). Amongst them, plant polyphenols have been shown to modulate molecular pathways in adipose pathology and help reverse obesity and obesity-related consequences (18). Since hydroxycinnamic acids are known to have radical scavenging activity, we hypothesized that SA might prevent adipocyte differentiation-induced dysfunction and oxidative stress. To test this hypothesis, we determined the *in vitro *anti-oxidant efficacy, anti-adipogenic and anti-ROS effect in cultured 3T3-L1 adipocytes. Additionally, the potential *in vivo* toxicity of SA on the *Drosophila melanogaster* insect model was evaluated.

## Materials and Methods

***Materials***

Dulbecco’s modified Eagle medium (DMEM), Dulbecco’s Phosphate Buffered Saline (DPBS), N-acetyl cysteine (NAC), 3-(4,5- dimethylthiazol-2-yl)-2,5-diphenyltetrazolium bromide (MTT), fatty-acid free bovine serum albumin (BSA), 2,2’-diphenyl-1-picrylhydrazyl (DPPH), 2,2’-azinobis (3-ethylbenzothiazoline-6- sulfonic acid) diammonium salt (ABTS), phenazine methosulphate (PMS), nitro blue tetrazolium chloride (NBT), Ellman’s reagent (DTNB; 5,5′-Dithiobis(2-nitrobenzoic acid)), Oil-Red-O (ORO), dimethyl sulphoxide (DMSO) and Hi-cDNA Synthesis Kit were obtained from HiMedia Laboratories, India. Newborn calf serum (NBCS) Foetal bovine serum (FBS) and antibiotic-antimycotic solution were obtained from Gibco®, Life Technologies, USA. Sinapic acid (SA), insulin, 3-isobutyl methylxanthine (IBMX), dexamethasone, 2’,7’-Dichlorofluorescin diacetate (DCFH-DA), and free glycerol reagent were obtained from Sigma (St. Louis, MO, USA). TRIreagent was obtained from Medox Biotech, India and iQ^TM^ SYBR^®^ Green Supermix from Bio-Rad (CA, USA). All reagents and chemicals used were of tissue culture and molecular biology grade.

***In vitro radical scavenging assays***

DPPH radical activity of SA and standard, ascorbic acid (10–1000 µmol) was measured with DPPH (1 mM). The solutions were incubated for 30 min at room temperature (RT) and optical density (OD) was measured at 517 nm in a spectrophotometer (19). ABTS radical scavenging assay was measured by preparing ABTS^+^ aqueous solution by mixing 7 mM ABTS and 2.4 mM potassium persulphate for 12 hr in the dark at RT. The solution was diluted to an absorbance of 1.2 by adding methanol at 734 nm. 1 ml of ABTS^+ ^solution and 50 µl of SA and standard were left to stand for 5 min and OD was recorded (20). Hydroxyl radical scavenging potential was measured using Fe^2+ ^-ascorbate-EDTA-H_2_O_2 _system (21). Reaction mixture containing 100 µl EDTA (1 mM), 10 µl FeCl_3 _(10 mM), 100 µl H_2_O_2 _(10 mM), 360 µl deoxyribose (10 mM), 330 µl phosphate buffer (50 mM, pH 7.4), 100 µl ascorbate and 100 µl of SA and standard was incubated for 1 hr at 37 °C. Equal volumes of TCA (10%) and TBA (0.5%) were added to the incubated mixture and development of MDA-TBA pink chromagen was observed at 535 nm. A nitric oxide scavenging assay was performed using the Griess method (22). SA and standard, ascorbic acid (10–1000 µmol), were mixed with 1 ml sodium nitroprusside (5mM) in phosphate buffer (0.1M, pH 7.4) and incubated for 2 hr at 25 °C. An equal volume of Griess reagent (1% sulphanilamide, 0.1% NED in 5% O-phosphoric acid) was added to the reaction mixture. OD was measured at 546 nm using a spectrophotometer. Percentage inhibition was calculated using the formula [(A_C_ – A_T_) / A_C_] x 100, where A_C _is the absorbance of the control and A_T _is the absorbance of the test. 

***Cell culture and differentiation***

3T3-L1 preadipocytes were purchased from the National Centre for Cell Sciences (Pune, India). Cells were cultured in high glucose (4.5 g/l) DMEM containing 10% NBCS at 37 °C in humidified 5% CO_2_. The cells were subcultured at 80% confluence and used to passage number 15 for experiments. In order to differentiate the cells for further experiment, the following protocol was followed (23). Two days post-confluent, cells (designated ‘day 0’) were placed in medium containing DMEM supplemented with 10% FBS and adipogenic induction cocktail, 0.5 mM IBMX (M), 1 μM dexamethasone (D), 10 μg/ml insulin (I) (MDI) (24). On day 2, the culture medium was replaced with DMEM supplemented with 10% FBS and 1 μg/ml insulin media (IM). The differentiating cells were fed with fresh IM every alternate day (days 2 to 8). On day 8, the mature adipocytes were shifted to basal medium (DMEM, 10% FBS) to complete the adipogenesis process. SA was added to the MDI and IM at different concentrations of 100, 250, 500, 750, and 1000 μmol/ml for the entire adipogenesis program (days 1 to 8). 

***Cell viability***

Preadipocyte (48h) and adipocyte viability (10 days) were tested by MTT assay (24). Cells (1x10^5^/ml) were seeded in a 96-well plate and treated with SA (100 – 1000 μmol/ml) and incubated at 37 °C, 5% CO_2_. At 48 hr or day 10, MTT (0.5 mg/ml) reagent was added and incubated for 4 hr. MTT was discarded and 100 μl of DMSO was added to dissolve the formazan crystals. OD was measured at 570 nm using a microplate reader (Multiskan™ FC Microplate Photometer, Thermo Fisher Scientific, MA, USA). Cell viability was expressed as a relative percentage compared with the negative control (100%). 

***Trypan blue staining***

For enumeration studies, 3T3-L1 preadipocytes undergoing differentiation with MDI were investigated on day 2 of the adipogenic program. MDI medium containing different concentrations of SA (100–1000 μmol/ml) was added to the cells for 48 hr. Following treatment, the cells were stained with 0.4% trypan blue solution and counted using a Neubauer hemocytometer (25). The undifferentiated cells were taken as control and compared with the treated cells.

***Oil red O (ORO) staining***

3T3-L1 preadipocytes (1x10^6^ cells/ml) were seeded in a 6-well plate and treated with SA (100–1000 μmol/ml) as per protocol. On day 10, the cells were fixed in 10% formalin in DPBS for 30 min and washed twice with DPBS. ORO stain (0.3% w/v ORO in 60% v/v isopropanol) (26) was added and incubated for 30 min at RT. Stained cells were observed under a phase-contrast microscope (Nikon Eclipse T*i*-S, Japan) at 200x magnification. The stain was eluted with 100% isopropanol and quantified at 520 nm using a spectrophotometer. 

***Triglyceride (TG) estimation***

SA treated (100–1000 μmol/ml) 3T3-L1 adipocytes were lysed with cell lysis buffer (0.1% Triton X-100 in DPBS) and the supernatant was collected following centrifugation at 12,000g for 15 min at 4 °C. TG was estimated using a commercially available kit (Lab Kit, India) as per manufacturer’s instructions. 

***Lipolysis assay***

Free glycerol levels in the culture medium were quantified on day 10. SA (100–1000 μmol/ml) in phenol red-free DMEM containing 10% BSA for 48 hr. The glycerol level was determined using free glycerol reagent (F6428) kit as per manufacturer’s protocol. 

***Intracellular ROS production***

3T3-L1 preadipocytes were differentiated in MDI with SA (100–1000 μmol/ml) and the extent of ROS (superoxides) production was determined by NBT assay (27). On day 10 after induction, the SA-treated cells are incubated with 0.2% NBT in DPBS for 90 min at 37 °C. Formazan was dissolved in 100% glacial acetic acid and OD was measured in a spectrophotometer at 570 nm (UV-1800, Shimadzu UV-spectrophotometer). For measuring DCFH-DA (for peroxides), 3T3-L1 preadipocytes were seeded in a 96-well plate at a density of 1x10^5 ^cells/ml and differentiated in MDI with SA(100–1000 μmol/ml) for 48 hr (28). ROS was measured by adding 20 µmol DCFH-DA to the cells and incubated at 37 °C for 30 min in a 5% CO_2_ incubator in the dark. Excess fluorescence was washed with DPBS and OD was measured in a spectrofluorometer at excitation of 480 nm and emission of 530 nm. 

***Intracellular glutathione (GSH) levels***

GSH levels in 3T3-L1 adipocytes were determined on day 10 following treatment with SA (100–1000 μmol/ml) (29). The differentiated cells were lysed in 1 ml of 0.2% Triton X-100 and 20 mM Tris-HCl buffer by sonicating thrice. The cell lysates were collected by centrifuging at 12,000g for 10 min at 4 °C. GSH was determined using DTNB. The reaction mixture of 1.8 ml consisted of 0.2M Na_2_HPO_4 _buffer, 40 µl of 10 mM DTNB, and 160 µl of cell lysate, incubated for 2 min. The OD was measured at 412 nm using a spectrophotometer.

***Superoxide dismutase (SOD) enzyme activity***

SOD levels were determined in 3T3-L1 adipocytes treated with SA (100–1000 μmol/ml) for 10 days (30). Briefly, 1.2 ml sodium pyrophosphate buffer (0.052 M, pH 8.3), 0.1 ml PMS (186 µM), 0.3 ml NBT (300 µM), and 0.1 ml cell lysate were added to a total volume of 3 ml. The reaction was initiated by adding 0.2 ml NADH (780 µM) and incubated for 90 sec at 30 °C. The reaction was stopped by adding 1 ml of glacial acetic acid and 4 ml n-butanol and shaken vigorously. Following a 10 min incubation period, the contents were centrifuged, and the OD was measured at 560 nm in a spectrophotometer. 

***Catalase (CAT) enzyme activity***

CAT activity was quantified on day 10 in 3T3-L1 adipocytes treated with SA (100–1000 μmol/ml) for (31). The reaction mixture consists of 1 ml of 0.01M phosphate buffer (pH 7.0), 0.5 ml 0.2 M H_2_O_2_, 0.4 ml of water and 0.2 ml of cell lysate. After 90 sec, the reaction was stopped by adding 2.0 ml of dichromate/acetic acid reagent (5% potassium dichromate with glacial acetic acid in 1:3 v/v ratio). The contents were heated for 10 min in a boiling water bath and OD was measured at 610 nm in a spectrophotometer. 

***RNA extraction and cDNA conversion***

Total RNA was extracted from 3T3-L1 preadipocytes treated with SA (100–1000 μmol/ml) in MDI except for negative control (undifferentiated). On day 10, total RNA was extracted using Medox Easy Total RNA Extraction Reagent (TRIzol) (Medox Biotech India Pvt. Ltd.). The isolated total RNA was quantified using a NanoDrop® ND-1000 spectrophotometer (Thermo Fisher Scientific, USA). 1000 ng RNA was reverse transcribed using oligo (dT) primer to cDNA using a conversion kit (Hi-cDNA Synthesis Kit, HiMedia Laboratories, India) according to the manufacturer’s protocol. 

***Quantitative real-time polymerase chain reaction (qPCR)***

qPCR was carried out using StepOnePlus™ Real-Time PCR System (Applied Biosystems, CA, USA) with iQ^TM^ SYBR^®^ Green Supermix (Bio-Rad, CA, USA). The PCR primers were purchased from Sigma-Aldrich, and the primer sequences are shown in Table 1. Amplification was performed in a 20 μl reaction mixture containing 1X SYBR Green PCR master mix, 300 nM each of forward and reverse primers, and 2 μl of target cDNA. The PCR conditions for thermal cycling were 95 °C for 10 min; followed by 40 cycles of denaturation at 95 °C for 15 sec, annealing, extension, and fluorescent reading at 60 °C for 60 sec. Relative amounts of mRNAs were calculated from the values of the comparative threshold cycle by using β-actin as an internal control. Negative control was included in each run, and the specificity of the amplification reaction was checked by melt curve analysis. The relative expression of genes was calculated using the 2^-Δct^ method. The RQ values were used to compare the gene expression of treated and control groups.

***Drosophila stock and culture***

The toxicity of SA was evaluated on the wild-type strain, Canton S procured from *Drosophila* stock center, University of Mysore. The flies were maintained on cornmeal agar containing 32% *w/v* corn flour, 14% w/v dextrose and sugar, 1% w/v agar-agar, 1% w/v yeast extract, and antifungal agent (propionic acid:orthophosphoric acid: benzoic acid) in glass bottles at a constant temperature of 25 °C in 12 hr light/dark cycle. Both sexes were used at random (32). 

***DNA fragmentation assay***

DNA fragmentation assay was performed in *Drosophila *(33). Flies bred in the ratio of 3 females to 1 male, and 5–7 day old adult flies were exposed to 1000 µmol SA, NAC, 100 µmol EMS (positive control), and sterile water (negative control) on potato media in the following manner. For parental (P) generation, the flies were exposed for 48 hr, and for filial (F_1_) generation, the flies were exposed to the compounds for 7 days. DNA was isolated from the whole body homogenate using the phenol:chloroform:isoamyl alcohol method (34). The isolated DNA was dissolved in 50 µl TE buffer (pH 8.0) and its quality was checked using a nanodrop spectrophotometer (NanoDrop® ND-1000, Thermo Fisher Scientific, USA). The DNA samples were electrophoresed on 2% agarose gel for 1 hr at 50V and the gel was viewed under a UV transilluminator and photographed in a gel documentation system. A no-objection certificate was granted for the *in vivo* study by the Institutional Animal Ethics Committee, SRIHER in accordance with OECD guidelines.

***Statistical analyses***

Results were expressed as mean± standard error of the mean (SEM) of values obtained from triplicates and analyzed using GraphPad Prism 8 software. Unpaired Student *t*-test was performed for single comparisons between two experimental groups. For all statistical analyses, a *P*-value less than 0.05 (*P*<0.05) was considered significant. 

## Results

***In vitro anti-oxidant assay***

The *in vitro *anti-oxidant activity of SA was evaluated by DPPH, ABTS, NO, and OH^*^. The concentration of SA required for 50% inhibition (IC_50_) is presented (Table 2). The radical scavenging activity of SA showed concentration-dependent anti-oxidant activity (*P*<0.001).

***Viability of 3T3-L1 cells***

**Figure 2:**
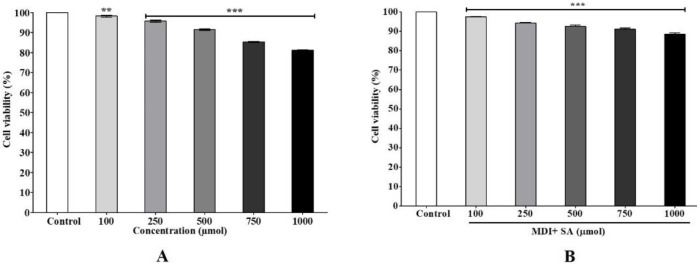
MTT assay. Cell viability of SA-treated cells at A) 48 hr (preadipocytes) and B) 10 days (adipocytes). Data are represented as mean±SEM of three independent experiments. *** *P*<0.001, Student's t-test compared with control (0 μmol). MDI: IBMX + DEX + I

SA at concentrations of 100, 250, 500, 750, and 1000 µmol/ml was treated to 3T3-L1 cells for 48 hr (preadipocyte) and 10 days (adipocyte), and the cell viability was measured**. **Over 80% cell viability was observed in presence of SA at 48 hr (Figure 2A) and >80% viability at 10 days (Figure 2B) compared with the control (100%). 

***SA inhibits lipid accumulation***

**Figure 3:**
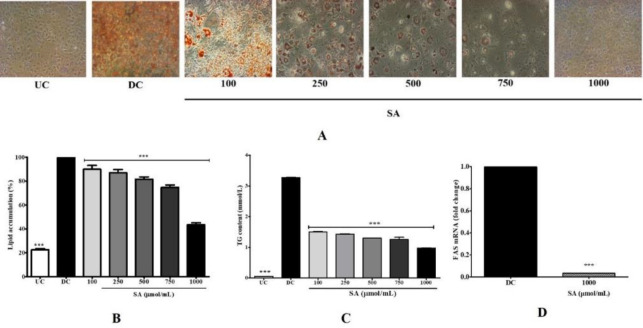
Effects of SA on the lipid accumulation (A, B) and TG levels (C) of 3T3-L1 cells. (A) Cells were cultured with or without SA (100–1000 μmol/ml). Cells were then stained with ORO staining solution and adipocytes were visualized under a microscope (200x). (B) Stained lipid droplets were solubilized with isopropanol and the absorbance was read at 520 nm. (C) Cellular TG content was measured using a commercial TG assay kit. (D) Expression of FAS messenger RNA levels in cells treated with 1000 μmol SA was measured by qRT-PCR analysis. Data are represented as mean±SEM of three independent experiments. *** *P*<0.001, Student's t-test compared with DC (0 μmol)

UC: undifferentiated control; DC: differentiated control; NAC: N-acetyl cysteine; SA: sinapic acid; TG: triglyceride; ORO: oil-Red-O

Lipid accumulation in 3T3-L1 adipocytes treated with various concentrations of SA was determined with ORO dye. As shown in Figure 3A, the amount of lipid accumulation decreased as the concentration of SA increased. On quantification, consistent with the staining, a significant dose-dependent decrease in lipid content was observed (Figure 3B). In the DC, the intracellular lipids accumulated at high levels compared with the preadipocyte undifferentiated cells (UC). The level of lipids was markedly reduced by 11, 13, 19, 26, and 57% (*P*<0.001) correspondingly, upon treatment with SA (100, 250, 500, 750, and 1000 μmol), demonstrating inhibition of lipid accumulation during differentiation of adipocyte cells. Additionally, TG contents were increased in DC but decreased 3-folds at 1000 μmol SA treatment (Figure 3C). The above results were in tandem with the level of FAS mRNA, a key enzyme of lipid biosynthesis. There was a 9-fold increase in DC, and almost completely inhibited following 1000 μmol SA treatment (Figure 3D). 

***SA inhibits MCE and early adipogenesis***

**Figure 4:**
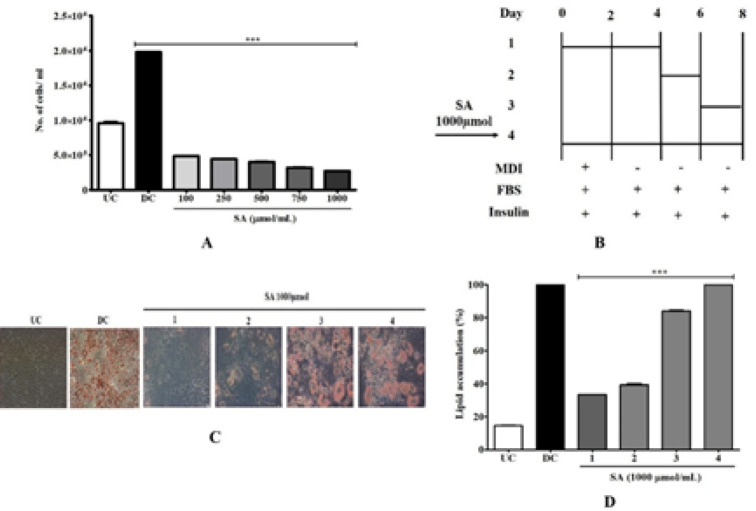
Effect on MCE by SA in 3T3-L1 cells. A) Differentiating adipocytes were treated with SA for 48 hr and the cell numbers were counted. B) Effect of SA on the stages of adipocyte differentiation. Fully confluent preadipocytes were initiated to differentiate by MDI cocktail with or without SA (1000 µmol/ml) for the indicated times. After 8 days, the differentiated cells were subjected to (C) ORO staining and (D) quantification of absorbance at 520 nm. Data are represented as mean±SEM of three independent experiments. *** *P*<0.001, Student's t-test compared with DC (0 μmol)

UC: undifferentiated control; DC: differentiated control; SA: sinapic acid; MCE: mitotic clonal expansion; ORO: oil-Red-O

The extent of mitotic clonal expansion (MCE) was quantified by counting SA-treated 3T3-L1 cells. The number of preadipocytes increased 2-fold from day 0 to 2 of differentiation in DC (differentiated control) (Figure 4A). In contrast, SA inhibited cell proliferation and only increased by 0.3 folds on day 2 compared with DC and UC (*P*<0.001). To identify the differentiation phase affected by SA, the differences between treatment periods were examined. Time points were divided into early adipogenesis (days 0–4), the middle stage (days 4–6), and the terminal stage (days 6–8). During adipogenesis, we treated cells with 1000 µmol SA at each time point (Figure 4B). Low lipid accumulation was observed when SA was treated at early adipogenic stages (days 0–4) (*P*<0.001) (Figure 4C). Treatment with SA after day 4 showed negligible effects compared with DC (Figure 4D). This indicates that SA suppressed lipid accumulation by inhibiting the early adipogenic stage.

***SA promotes lipolysis in 3T3-L1 adipocytes***

**Figure 5.**
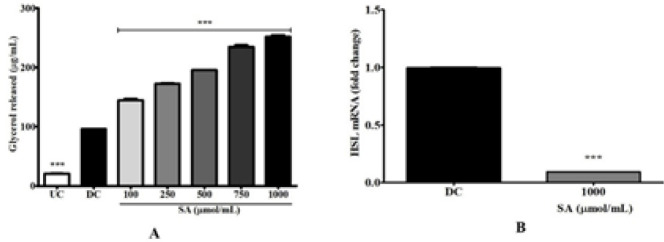
Lipolysis in SA-treated 3T3-L1 cells. Preadipocytes were differentiated into adipocytes for 10 days in MDI and various concentrations of SA (100−1000 μmol/ml). (A) Measurement of glycerol released and (B) Expression of HSL messenger RNA levels in cells treated with 1000 μmol SA were measured by qRT-PCR analysis. Data are represented as mean±SEM of three independent experiments. *** *P*<0.001, Student's t-test compared with DC (0 μmol)

UC: undifferentiated control; DC: differentiated control; SA: sinapic acid; HSL: hormone-sensitive lipase

Glycerol release from the SA-treated cells increased in a concentration-dependent manner (*P*<0.001). At 1000 μmol SA, a 200% increase was observed compared with DC (Figure 5A). Furthermore, the effect of SA on mRNA expression of the lipolysis gene, HSL was evaluated with qPCR. In Figure 5B, contrastingly, the cells cultured in MDI containing 1000 μmol SA decreased the mRNA levels of HSL genes (*P*<0.001).

***SA affects ROS production in 3T3-L1 adipocytes***

**Figure 6:**
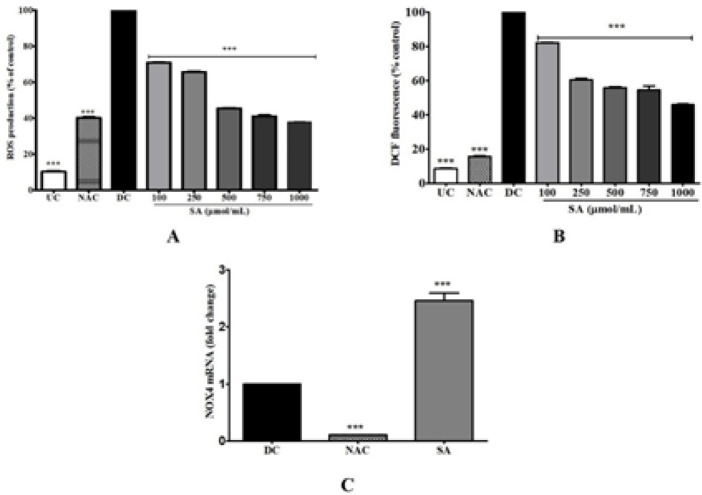
Effect of SA on ROS production in 3T3-L1 adipocytes. A) NBT assay; B) DCF-DA staining. The 3T3-L1 adipocytes were exposed to SA (100−1000 μmol/ml) and NAC (1000 μmol/ml), for 48 hr, and absorbance was quantified. (C) Expression of NOX4 messenger RNA levels was measured by qRT-PCR analysis. 3T3-L1 adipocytes were cultured with SA (1000 μmol/ml) and NAC (1000 μmol/ml) for 10 days. Data are represented as mean±SEM of three independent experiments. *** *P*<0.001, Student's t-test compared with DC (0 μmol)

UC: undifferentiated control; DC: differentiated control; NAC: N-acetyl cysteine; SA: sinapic acid

Differentiation significantly increased ROS generation in cells whereas treatment with 1000 μmol NAC, an effective cellular anti-oxidant, reduced ROS. NBT assay detects peroxides in cells undergoing oxidative stress (Figure 6A). Addition of SA to MDI during adipogenesis resulted in decreased ROS generation in cells in a concentration-dependent-manner (*P*<0.001). Similarly, doses of SA showed amelioration effect of ROS by DCF-DA which showed SA significantly (*P*<0.001) protects cells from ROS generation (Figure 6B). However, the inhibitory effect of SA on ROS inhibition was not reflected in the regulating enzyme, NOX4 (Figure 6C). An up-regulation in NOX4 mRNA levels was observed at 1000 μmol SA treatment, whereas NOX4 (1000 μmol) mRNA was down-regulated (*P*<0.001).

***SA increases GSH and anti-oxidant enzymes in 3T3-L1 adipocytes***

**Figure 7:**
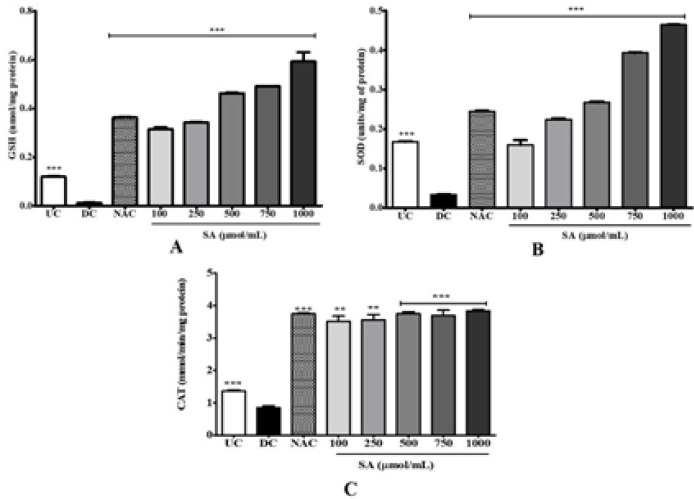
Effect of SA on GSH and anti-oxidant enzyme activities (SOD and CAT) in 3T3-L1 cells. 3T3-L1 adipocytes were differentiated in MDI supplemented with SA (100−1000 μmol/ml) and NAC (1000 μmol/ml) for 10 days, and the levels of (A) GSH; (B) SOD; and (C) CAT were estimated. Data are represented as mean±SEM of three independent experiments. *** *P*<0.001, Student's t-test compared with DC (0 μmol)

UC: undifferentiated control; DC: differentiated control; NAC: N-acetyl cysteine; SA – sinapic acid); GSH: intracellular glutathione; SOD: superoxide dismutase; CAT: catalase

The changes in intracellular GSH, the major intracellular anti-oxidant were quantified in 3T3-L1 adipocytes on day 10. As shown in Figure 7A, treatment with SA (100–1000 μmol/ml) caused in significant (*P*<0.001) concentration-dependent manner increase in GSH levels compared with DC. The changes in the intracellular anti-oxidant enzymes, SOD (Figure 7B), and CAT were also examined (Figure 7C). Treatment with SA induced significant (*P*<0.001) and concentration-dependent increases in activity compared with DC. 

***Suppression of adipogenic transcription factor expression by SA***

**Figure 8.**
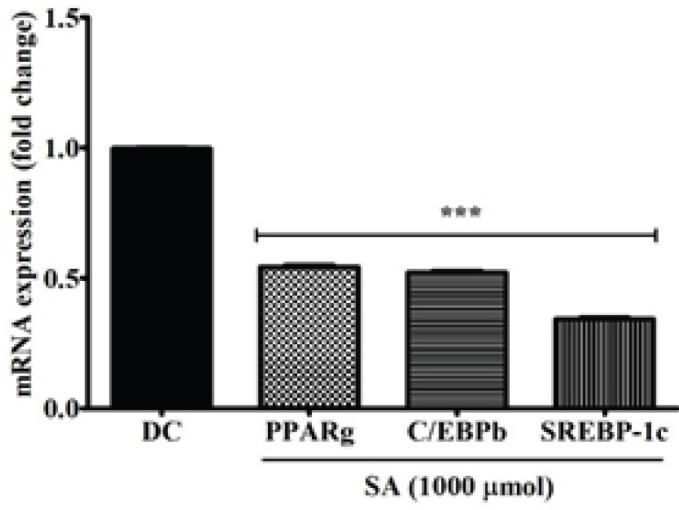
Effect of SA on adipogenic gene expression of PPARγ, CEBPβ, SREBP1c. 3T3-L1 cells were treated with 1000 μmol/ml SA for 10 days in an MDI medium. Relative expression levels of PPARγ, CEBPβ, and SREBP1c were quantified by qRT-PCR. Data are represented as mean±SEM of three independent experiments. *** *P*<0.001, Student's t-test compared with DC (0 μmol)

DC: differentiated control; SA: sinapic acid; MDI: IBMX, dexamethasone, and insulin

The effects of SA (100−1000 μmol/ml) on the expression of adipogenic transcription factor genes such as PPARγ, C/EBPβ, and SREBP-1c were evaluated by quantitative RT-PCR. In Figure 8, the expressions of PPARγ, C/EBPβ, and SREBP-1c were enhanced in DC (treated with only MDI) due to adipogenesis. In adipocytes treated with 1000 µmol of SA, the mRNA levels of PPARγ and C/EBPβ decreased 50-fold and SREBP-1c 70-fold, significantly (*P*<0.001). 

***DNA fragmentation assay***

The isolated DNA was quantified and analyzed on 2% agarose. The intensity of DNA damage was higher in flies treated with 100 µmol EMS indicating shearing, apoptosis, and subsequent cell death. Whereas, no shearing or fragmentation was observed in flies treated with 1000 µmol SA (both parental and filial (F_1_) generation), NAC, and control. 

In the present study, we treated the 3T3-L1 cells with saponin of KD-D during differentiation and found

**Table 1:** List of primers used in RT-PCR study

Primer name	Nucleotide sequence (5’ to 3’)		Product size (bp)	Accession no:
**β-actin**	Sense	AATGTAGTTTCATGGATGCC	430	NM_007393.5
	Antisense	CCAGATCATGTTTGAGACCT		
**PPARγ**	Sense	CCCTGGCAAAGCATTTGTAT	164	XM_036165927.1
	Antisense	GAAACTGGCACCCTTGAAAA		
**C/EBPα**	Sense	ATCCCAGAGGGACTGGAGTT	373	NM_001287514.1
	Antisense	AAGTCTTAGCCGGAGGAAGC		
**SREBP-1c**	Sense	TCATGCCCTCCATAGACACA	215	XM_006532716.4
	Antisense	GCTCAAAGACCTGGTGGTG		
**FAS**	Sense	CTGAGATCCCAGCACTTCTTGA	101	XM_030245556.1
	Antisense	GCCTCCGAAGCCAAATGAG		
**HSL**	Sense	ACAGTGCAGGTGGGAATCTC	242	XM_030242180.1
	Antisense	GCCTAGTGCCTTCTGGTCT		
**NOX4**	Sense	GAAGCCCATTTGAGGAGTCA	427	XM_006508010.4
	Antisense	GGGTCCACAGCAGAAAACTC		

MTT: 3-(4,5- dimethylthiazol-2-yl)-2,5-diphenyltetrazolium bromide SA: sinapic acid

**Figure 9:**
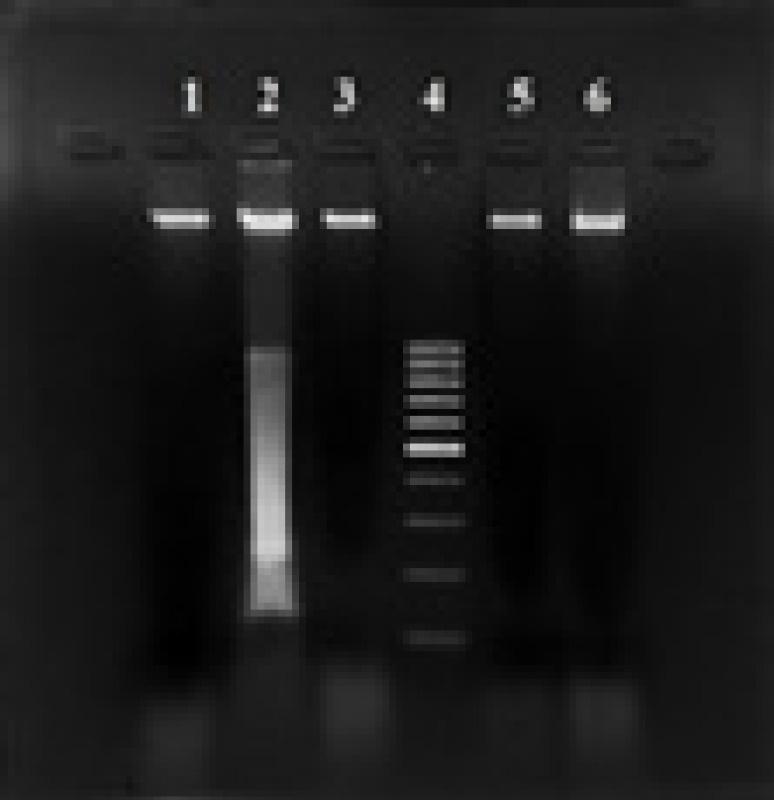
DNA fragmentation assay. The isolated whole genome of Drosophila was electrophoresed on 2% agarose gel. 1 – Control, 2 – 100 µmol EMS, 3 – 1000 µmol NAC, 4– 100 bp molecular ladder, 5 – 1000 µmol SA exposed parental generation, and 6– 1000 µmol SA exposed filial (F1) generation

EMS: Ethyl methanesulfonate; NAC: N-acetyl cysteine; SA: sinapic acid

## Discussion

Plant-based phytochemicals are gaining popularity as the subject of many research studies, as most anti-obesity medicines have adverse effects. Obesity is defined by the formation of lipid droplets and development of preadipocytes into mature adipocytes. Excessive preadipocyte development causes aberrant adipose tissue expansion and accumulation which are linked to obesity. Thus, potent inhibitors of preadipocyte development and differentiation may have therapeutic and/or preventative anti-obesity effects. Natural compounds with anti-obesogenic potential can be used as an alternative therapeutic option in treating obesity. The results of the study support the hypothesis that SA modulates adipogenesis *via* its anti-adipogenic, anti-lipogenic, and anti-oxidant activity in 3T3-L1 adipocytes. 

To begin, in this study, we demonstrated that SA suppressed preadipocyte cell expansion without inducing cell toxicity. Growth-arrested 3T3-L1 preadipocytes induced to differentiate re-enter the cell cycle and undergo several rounds of mitosis called MCE, which is crucial for determining the transcriptional cascade of adipogenesis. Upon exiting the cell cycle, they lose their fibroblastic morphology and acquire the metabolic characteristics of a fully mature adipocyte. Likewise, addition of SA at the early stage of adipocyte differentiation (days 0 to 4) showed maximal prevention in lipid droplet formation (Figure 4C). This result suggests that the anti-adipogenic function of SA begins from the early stage of adipocyte differentiation. Blocking DNA replication by inhibiting MCE thus preventing differentiation has been documented with phytocompounds like curcumin (35), rehmannia (25), and piceatannol (36). 

SA decreased lipid and TG levels in 3T3-L1 cells during adipocyte development and stimulated the release of glycerol in 3T3-L1 adipocytes (Figure 2). This discovery prompted us to study its impact on expression of the lipogenesis-related gene, FAS. SA significantly suppressed the expression of FAS, thereby contributing to reduced lipid production, storage, and accumulation (Figures 3B & C). FAS is a lipid anabolic gene that catalyzes synthesis of palmitate from acetyl-CoA and malonyl-CoA into long-chain saturated fatty acids (37). The levels of FAS were found to be significantly down-regulated with 1000µmol SA but highly up-regulated in DC (Figure 3D), suggesting that SA negatively regulates fatty acid synthesis by down-regulating FAS. Therefore, our results demonstrate that SA inhibits *de novo* triglyceride synthesis and ectopic fat deposition. Hydrolysis of TG releases glycerol and free fatty acid from adipocytes. Glycerol in the media was found to be significantly higher with SA treatment (Figure 5A). Contrarily, mRNA expression of HSL, the rate-limiting enzyme in diacylglycerol metabolism, was found to be down-regulated compared with DC (Figure 5B). This could be due to the variation in phosphorylation following post-transcription. In murine adipocytes, HSL is phosphorylated at three serine residues namely 563, 659, and 660 by PKA activity, leading to translocation of HSL onto the surface of lipid droplets (38). But phosphorylation of HSL by AMP-activated protein kinase of Ser^565 ^(39) and by Akt-mediated phosphorylation of Ser^273^ by activation of phosphodiesterase 3B (40) mitigates PKA activity and prevents HSL activation and lipolysis. Therefore, down-regulation of HSL in this instance could be due to any of these mechanisms. 

Adipogenesis requires a network of transcription factors that contribute to sequential gene expression for adipocyte differentiation and maturation. PPARγ, the master regulator of adipogenesis, is essential for differentiation (41). This leads to induction of C/EBPα and SREBP-1c expression, which successively activate other adipocyte genes responsible for terminal differentiation (42). Our compound SA at 1000 µmol significantly inhibited the expression of PPARγ, C/EBPα, and SREBP-1c. C/EBPα is expressed immediately after exposure to MDI, whereas PPARγ and C/EBPα are acquired following 36–48 hr of exposure. Therefore, SA inhibiting the early stage of differentiation is in accordance with the repressed expression of C/EBPα and PPARγ. The down-regulation of SREBP-1c expression at the transcriptional and translational levels is evident in the reduced expression of their downstream target genes, including FAS. Many compounds have been described as inhibiting adipogenesis via PPARγ, C/EBPα, and SREBP-1c regulation (43–45). Thus, considering that SA largely reduced mRNA expressions of PPARγ, C/EBPα, SREBP-1c, and FAS during 3T3-L1 preadipocyte differentiation, herein, it is evident that the anti-adipogenic effect is closely linked to their reduced expression.

Several phytochemicals are known to inhibit the anti-adipogenic effect through their anti-oxidant activity (46). Herein, the *in vitro* anti-oxidant activity of SA was demonstrated through DPPH, ABTS, NO, and OH radical scavenging activity (Table 2). The effective anti-oxidant property of SA is due to its aromatic phenolic ring (Figure 1) that delocalizes and stabilizes unpaired electrons within its ring structure, thereby acting as free-radical scavengers (47). Increase in the number of methoxy substitutions in positions ortho to the OH in monophenols like SA increases greatly and enhances the electron-donating properties in the 4- or 4’-position (48). The other hydroxycinnamic acids, such as p-coumaric, caffeic, and ferulic are also known to be excellent anti-oxidants. The anti-oxidant activity of SA in 3T3-L1 adipocytes was examined to further determine its anti-oxidant ability. Intense adipogenesis in obesity is strongly correlated with oxidative stress which leads to production of ROS (49). ROS in adipocytes is generated by NOX4 during adipogenesis causing insulin resistance and cell damage (50). NOX4 is especially expressed in adipocytes and acts as a switch between proliferation and differentiation of adipocytes (51). Adipogenesis leads to low levels of endogenous anti-oxidant enzymes such as SOD, CAT, and GSH (27). Therefore, inhibiting ROS production in adipocytes can be a potential target for improving obesity. SA effectively reduced ROS levels during adipogenesis (Figures 6A & B) compared with DC and NAC. This could be due to the effective anti-oxidant ability of SA. As shown in Figure 7A, SA increased GSH, SOD, and CAT in a concentration-dependent manner compared with DC. Previous studies have shown that phytocompounds like resveratrol (52), esculetin (53), and dibenzoylmethane (54) reduce ROS levels in murine adipocytes. To gain further insight into the mechanism of SA-mediated ROS inhibition, the mRNA expression levels of NOX4, the major pro-oxidant in 3T3-L1 adipocyte was studied (Figure 6C). The NOX4 mRNA level of NAC-treated adipocytes was greatly increased whereas a decrease was observed in cells treated with 1000 µmol SA. Inhibition of ROS generation in the NBT and DCF-DA assay is not completely reflected by the NOX4 gene. This suggests a much more complex ROS regulation mechanism with involvement with other genes that control ROS production in adipocytes. Therefore, SA can inhibit adipogenesis-induced intracellular ROS production through its anti-oxidant activity and by up-regulating the cellular anti-oxidant defense mechanisms. 

The fruit fly, D. melanogaster is a reliable model for assessment of toxicity of food or chemical structures (55) and an alternative method to the use of the animal model (56, 57). Since the eukaryotic genome of Drosophila has more than 80% homology with disease-related loci in humans, the results obtained are highly specific and translational (58). DNA shearing and fragmentation are indicative of the first-line toxicity of a compound (59). In the present study, SA did not show any DNA shearing or fragmentation in both parental and F1 generations. Whereas, DNA smearing was observed with EMS treatment, which is indicative of activation of caspase-3 (60). This suggests that SA is non-toxic to the eukaryotic genome during long-term application of the compound. 

## Conclusion

We present *in vitro* the anti-adipogenic potential of SA by reducing the preadipocyte clonal population and inhibiting adipocyte differentiation by down-regulating adipogenic transcription factors and lipogenesis. SA showed improved lipolysis and cellular anti-oxidants thereby preventing intracellular ROS accumulation. Also, SA was a non-toxic Drosophila model on long-term exposure. These results suggested that SA could be used as a possible candidate for development of clinically effective anti-obesity agents. 

## Authors’ Contributions

SA Provided study conception and design, prepared the draft manuscript, and helped with visualization. CMJ Helped with data processing and collection and performed experiments. CMJ and SA Prepared the draft manuscript and helped with visualization. CMJ analyzed and interpreted the results. CMJ and SA Critically revised or edited the article. SA Approved the final version to be published. SA Supervised, and helped with funding acquisition.

## Conflicts of Interest

The authors declare no conflicts of interest.
